# Arterial enhancing local tumor progression detection on CT images using convolutional neural network after hepatocellular carcinoma ablation: a preliminary study

**DOI:** 10.1038/s41598-022-05794-8

**Published:** 2022-02-02

**Authors:** Sanghyeok Lim, YiRang Shin, Young Han Lee

**Affiliations:** 1 Department of Radiology, Research Institute of Radiological Science, and Center for Clinical Imaging Data Science (CCIDS), Yonsei University College of Medicine, Seoul, Korea; 2 Present Address: Department of Radiology, SoonChunHyang University Bucheon Hospital, SoonChunHyang University College of Medicine, Bucheon-si, Gyeonggi-do Korea

**Keywords:** Oncology, Cancer imaging

## Abstract

To evaluate the performance of a deep convolutional neural network (DCNN) in detecting local tumor progression (LTP) after tumor ablation for hepatocellular carcinoma (HCC) on follow-up arterial phase CT images. The DCNN model utilizes three-dimensional (3D) patches extracted from three-channel CT imaging to detect LTP. We built a pipeline to automatically produce a bounding box localization of pathological regions using a 3D-CNN trained for classification. The performance metrics of the 3D-CNN prediction were analyzed in terms of accuracy, sensitivity, specificity, positive predictive value (PPV), area under the receiver operating characteristic curve (AUC), and average precision. We included 34 patients with 49 LTP lesions and randomly selected 40 patients without LTP. A total of 74 patients were randomly divided into three sets: training (n = 48; LTP: no LTP = 21:27), validation (n = 10; 5:5), and test (n = 16; 8:8). When used with the test set (160 LTP positive patches, 640 LTP negative patches), our proposed 3D-CNN classifier demonstrated an accuracy of 97.59%, sensitivity of 96.88%, specificity of 97.65%, and PPV of 91.18%. The AUC and precision–recall curves showed high average precision values of 0.992 and 0.96, respectively. LTP detection on follow-up CT images after tumor ablation for HCC using a DCNN demonstrated high accuracy and incorporated multichannel registration.

## Introduction

Typically, hepatocellular carcinoma (HCC) shows arterial enhancement and delayed washout on dynamic contrast-enhancement imaging studies, such as computed tomography (CT), magnetic resonance imaging (MRI), and ultrasonography (US)^1^. This finding enables non-invasive diagnosis without biopsy and provides reliable and reproducible imaging data to diagnose and detect tumor recurrence during follow-ups after treatment^2^.

Image-guided tumor ablation is a curative method comparable to surgery for patients with early-stage HCC^3^. The ablation zone is visualized as a hypodense or heterogeneous hyperdense region on an unenhanced CT without enhancement after contrast administration. Follow-up imaging study is crucial to detect recurrence because recurrent HCC is not a rare event^4^. Moreover, early detection and appropriate treatment enables effective treatment.

Deep convolutional neural networks (DCNNs) are deep learning neural networks in which multiple hidden layers are trained to perform particular tasks^5^. They have been used successfully in medical fields including radiology^5,6^. A DCNN extracts low- to high-level features from images and uses them to select the most important features for solving a specific task, such as classification, detection, and segmentation^7^. We hypothesize that the DCNN may be able to detect small enhancing lesions on CT, that is, local tumor progression (LTP). Herein, we propose a novel three-dimensional (3D) multichannel DCNN framework for false positive reduction and to increase the conspicuity of the region of interest. Hence, the purpose of this study was to evaluate the performance of the DCNN in detecting LTP after tumor ablation for HCC on follow-up arterial phase CT images.

## Results

### Participants

Among the 184 patients who underwent complete ablation for treatment-naïve single HCC, 26 patients were excluded from the analysis owing to intrasegmental aggressive recurrence, liver transplantation during the follow-up period, and unavailable follow-up CT images. We recruited 34 patients with LTP (male: female = 29:5, age, 63.3 ± 8.1; range 45–84 years). Among the 124 patients without LTP, we randomly selected 40 patients (male: female = 31:9; age, 65.7 ± 11.8; range 30–86). Therefore, 74 patients were enrolled (mean age, 64.6 ± 10.3; range 30–86; 60 men [mean age, 64.0 ± 10.0; range 30–85] and 14 women [mean age, 67.3 ± 11.6; range 45–86]). The participants’ demographic characteristics are shown in Table 1.

**Table 1:** Clinical characteristics of the 74 study patients.

	LTP group (n = 34)	No LTP group (n = 40)	*p* value
Sex (male:female)	29:5	31:9	0.40
Age (years)	63.3 ± 8.1	65.7 ± 11.8	0.31
LTP size (cm)	1.2 ± 0.5	NA	NA
Time interval between ablation and CT (days)	255 ± 189	414 ± 90	NA
Time interval between ablation and last follow up (days)	NA	734 ± 166	NA
**Etiology of liver disease**			
HBV	25	23	0.37
HCV	1	5	
Alcohol	5	6	
Others	3	6	

LTP, local tumor progression; CT, computed tomography; NA, not applicable; HBV, hepatitis B virus; HCV, hepatitis C virus.

### Diagnostic performance of 3D CNN

**Figure 1:**
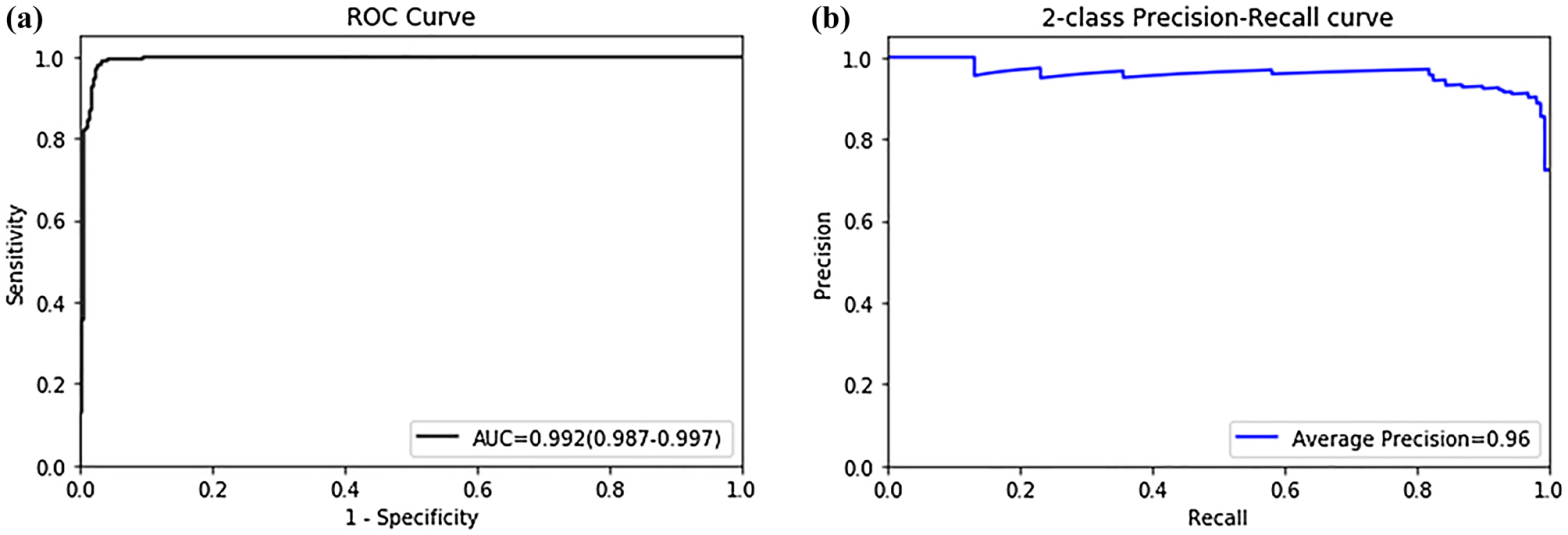
Receiver operating characteristic curve (**a**) and precision–recall curve (**b**) of LTP classification model.

When used with the test set (160 LTP positive patches, 640 LTP negative patches), our proposed 3D-CNN classifier demonstrated an accuracy of 97.59%. Table 2 summarizes the diagnostic performance of the 3D-CNN in classifying LTP using a 3D abdominal volume with a sensitivity of 96.88%, specificity of 97.65%, and PPV of 91.18%. We plotted the ROC curve in Fig. 1a, where the AUC of the ROC curve for the 3D-CNN model was 0.922 (95% CI 0.987–0.997). In addition to evaluating the accuracy of the model using the ROC curves, precision–recall curves were plotted, as shown in Fig. 1b, where our model yielded a high average precision value of 0.96. A high area under the precision–recall curve (average precision) suggests a high precision (low false-positive rate) and a high recall (low false-negative rate). Therefore, our prediction model incorporating multichannel registration and a 3D-CNN, can accurately detect LTP and distinguish it from normal structures other than LTP.

**Table 2:** Model performance on test datasets for classifying LTP.

Method	Value	95% CI
AUC	0.992	0.987–0.997
Sensitivity (%)	96.88	92.86–98.98
Specificity (%)	97.65	96.16–98.68
PPV (precision, %)	91.18	86.23–94.46
Accuracy	97.59	96.17–98.47
AP	0.96	

LTP, local tumor progression; CI, confidence interval; AUC, area under receiver operating characteristic curve; PPV, positive predictive value; AP, average precision.

## Discussion

Radiologic diagnosis techniques using artificial intelligence (AI) are being widely developed and used^8–10^. Because the detection and interpretation of enhancing lesions on follow-up imaging studies are essential for diagnosing HCC recurrence, in the current study, we use AI to focus on the detection of arterial enhancing lesions next to an ablation zone and distinguish them from LTP and other possible enhancing lesions, including normal vasculatures or arterioportal shunts.

Our study demonstrated that 3D multichannel DCNNs can detect small enhancing lesions, which are typical indicators of LTP on CT images. We integrated a 3D-DCNN model with multichannel registration to classify LTP regions. Deep learning with 3D-DCNNs can accurately classify LTP regions on liver CT images with an AUC of 0.992. Considering the radiologic imaging interpretation, we applied a 3D model to the liver CT images. We incorporated 3D models instead of 2D models to account for intraslice information, thereby reducing false positive rates, such as blood vessels, ablation zone proximity, and longitudinal passing. As a 3D-DCNN model with full-size CT images requires high-performance computing power and more computational memory, we avoided memory exhaustion using a relatively small input by patch extraction. DICOM images have full pixel ranges of 12 bits (4,096 shades of gray per pixel), which cannot be directly input to a DCNN model in the current technology. We transferred a CT image with a range of 12 bits with predefined window settings optimized for LTP pathologies to obtain an 8-bit grayscale image with enhanced conspicuity of LTP regions. To maximize the image contrast, we used a predefined window setting in three channels of the image: the liver, ablation, and tumor windows.

**Figure 2:**
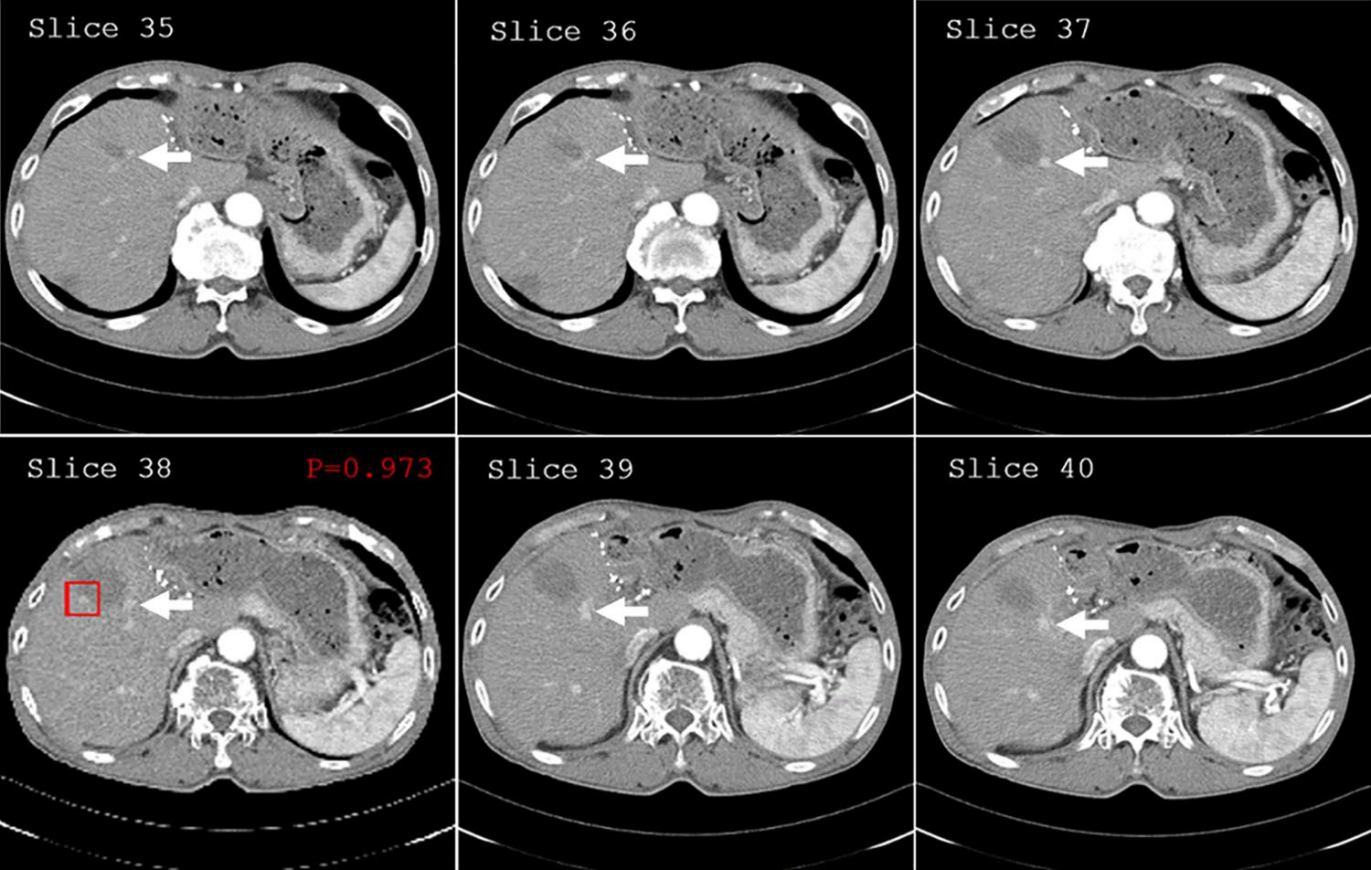
Prediction map for true positive predicted LTP region. In the prediction map, we report the rectangular region of interest on the axial slice of the whole CT scan, slide number, tumor prediction probability, as well as neighboring image slices included in the positive-predicted regions. LTP was detected (red rectangular), and hepatic vessels including hepatic artery and portal vein (arrows) were not indicated in the prediction map.

During classification, a prediction map was applied to generate bounding boxes to highlight the positively predicted regions to achieve rapid and accurate detection of LTP for users (Fig. 2). A dictionary storing (x,y,z)-center coordinates was retrieved based on prediction probabilities above the optimal threshold value that were selected using sensitivity and specificity. For each slide, we report the rectangular region of interest on the axial slice of the whole CT scan, slide number (z), and tumor prediction probability, as well as neighboring image slices included in the positive-predicted patch.

**Figure 3:**
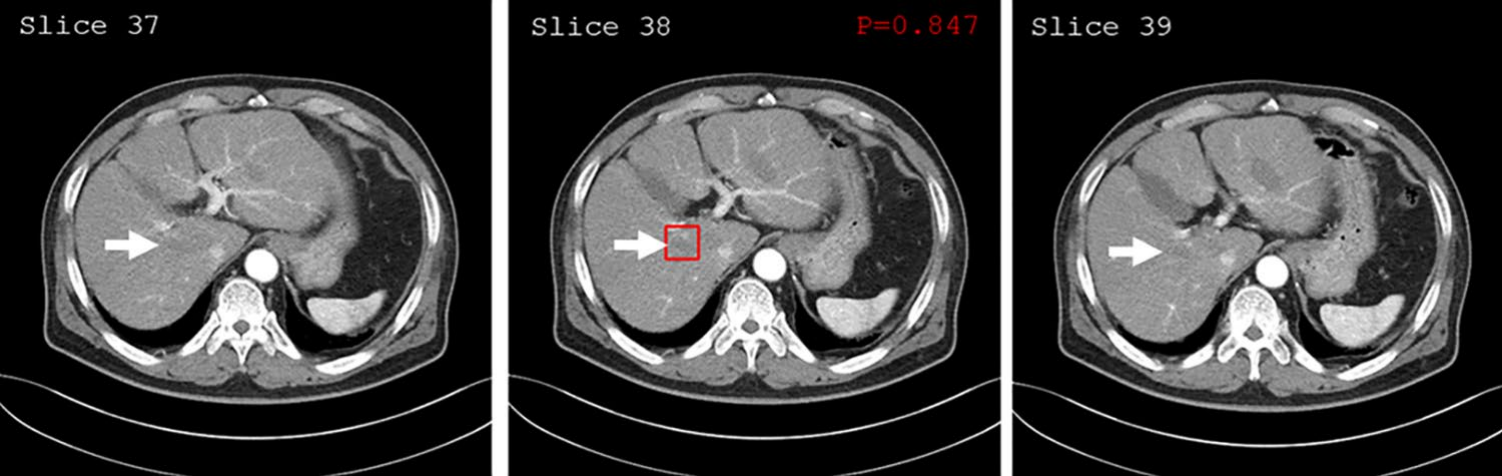
Prediction map for false-positive-predicted LTP region. LTP was detected (red rectangular) on the prediction map. However, we discovered that the detected region was far from the ablation zone. The unenhanced right hepatic vein (arrows), next to the contrast-enhanced hepatic vessels, was regarded as an ablation zone from artificial intelligence.

Two independent radiologists reviewed two false-positive and three false-negative prediction regions within the liver. One false-positive case (Fig. 3) showed an enhancing nodular lesion next to the hypo-attenuating region, which was confirmed to be an unenhanced right hepatic vein next to the contrast-enhanced hepatic vessels, and not an ablation zone. The other lesion was confirmed to be an arterioportal shunt at the periphery of the ablation zone (not shown). Although the 3D DCNNs reduced the false-negative prediction of the model, examples of false-negative cases were primarily associated with large lesions. The mean diameter of the three false-negative cases on the axial images was 1.9 cm (ranging from 1.1 to 2.6 cm), which was larger than the initial patch size (32 × 32).

The diagnostic performance of LTP using MRI or contrast-enhanced US (CEUS) after ablation for HCC is higher than that using CT. However, medical resources and costs are an issue in surveillance settings. Mostly, patients treated for HCC have chronic liver disease, including liver cirrhosis, and must be evaluated not only for LTP, but also for whole abdomen metastasis and complications associated with portal hypertension, including varices and ascites. Thus, we used CT as a surveillance tool and used MRI or CEUS as a confirmatory tool. We hypothesize that it might be possible to extrapolate the current DCNN on MRI and CEUS. However, in this preliminary report, there were too few cases with MRI or CEUS performed. Further well-designed prospective studies are warranted to verify this finding.

Our study has a few limitations. First, Tour models were trained and tested on a small dataset of only 74 patients. Therefore, to obtain a better generalizability of the proposed method, we must increase the number of participants, including external validation collected by case–control design, diagnostic cohort design, or multi-institution external test in the future^11^. Second, in a retrospective study design, we used a two-vendor CT dataset. However, the effects of different CT machines would be limited because our model focused on detecting an enhancing nodule next to an ablation zone. We overcome these expected drawbacks using multichannel images comprising different window levels/widths. Subsequently, we incorporated images of different window settings, which were clinically used, by channel registration and then used them for LTP prediction without considering optimal window settings for deep learning models. Third, we extracted random patches to be used as our 3D DCNN input; therefore, region proposal methods or methods that identify discriminative patches should be investigated in the future. Finally, only the arterial-phase images were trained in our model. Although we trained a limited number of images, our primitive model showed the feasibility of LTP candidates in patients who had undergone tumor ablation. Extended models, including all image phases, should be developed to achieve better performances.

In conclusion, LTP detection on follow-up CT images after local ablation for HCC using a DCNN demonstrated high accuracy; multichannel registration was incorporated and the 3D-CNN can accurately predict and differentiate LTP candidates from others, including normal vasculature. This will be a useful tool for early detection of possible LTP candidates on follow-up CT images by reducing the effort of finding enhancing lesions for radiologists and allowing only delayed washouts to be checked.

## Methods

### Ethical considerations

This retrospective study from a single tertiary center was approved by the Institutional Review Board of Yonsei University’s Health System (IRB No: 4–2019-1123) and was granted a waiver of written informed consent for use of data. All methods were performed in accordance with the relevant guidelines and regulations. The study was conducted in compliance with the principles of the Declaration of Helsinki. The authors have complete control of the data and the information submitted for publication.

### Participants

**Figure 4:**
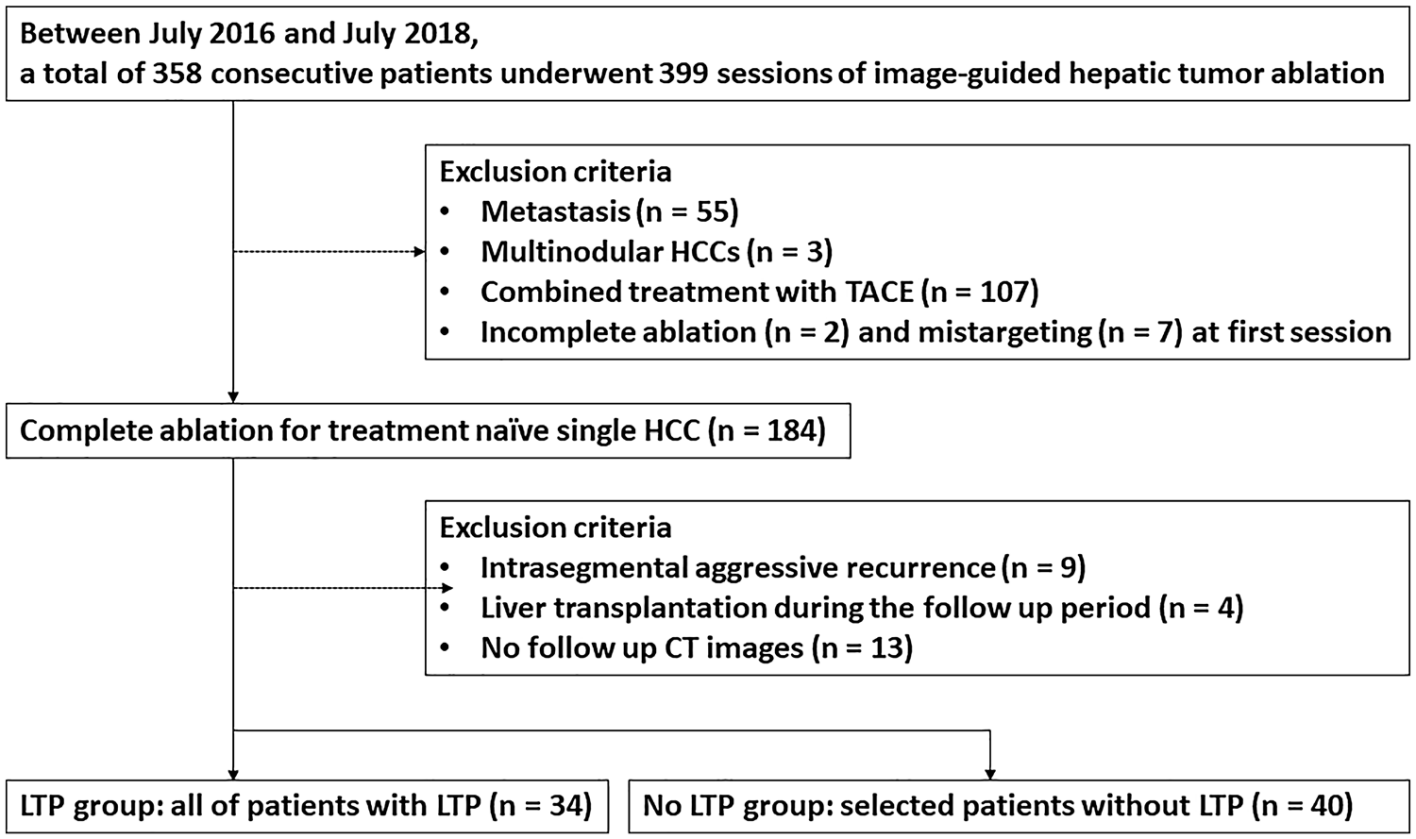
Flow diagram of study population.

One radiologist (S Lim, a certified abdominal radiologist with more than 10 years of experience in abdominal imaging and more than 7 years of experience in image-guided tumor ablation for hepatic tumors) reviewed the electronic medical records as well as picture archiving and communication system records. We identified 358 consecutive patients who underwent 399 sessions of image-guided hepatic tumor ablation, including radiofrequency ablation (RFA) or microwave ablation (MWA), between July 2016 and July 2018 (Fig. 4). Eligibility criteria were designed to include the technical success achieved in treatment-naïve single HCC. Technical success was defined as whether the index tumor was covered completely by the ablation zone, according to the protocol, and was assessed immediately following contrast-enhanced CT^12^. LTP was defined as the appearance of new tumors at the ablative margin after local eradication of all tumor cells with ablation on follow-up imaging studies. For the LTP group, eligibility criteria were designed to include radiologically identified LTP during the follow-up period. Patients diagnosed with intrasegmental aggressive recurrence, without CT follow-up data, and those who underwent liver transplantation during the follow-up period were excluded^13^. We compared the ablation zone lesion by lesion on follow-up imaging studies of each patient for two years after ablation. All patients underwent follow-up studies including contrast-enhanced CT or MRI examinations, chest radiography, and laboratory tests including serum alpha-fetoprotein (AFP) one month after initial RFA, after three months, and thereafter every six months during the first two years. For the no LTP group, among 124 patients without evidence of LTP during the follow-up period, 40 patients were selected by simple random sampling. No LTP was defined as the absence of lesions showing arterial enhancement and delayed washout on the follow-up image (one more follow-up image provided data for training and test sets); additionally, no elevation of tumor markers was observed. Two radiologists (S Lim and YH Lee) reviewed all CT images of both LTP and no-LTP groups in consensus to check whether the images of patients met the eligibility criteria.

### CT image acquisition

CT was performed using one of three 64-, 128-, or 256-channel multidetector CT scanners (Siemens Healthineers, Erlangen, Germany; GE Healthcare, Waukesha, WI, USA). The routine four-phase dynamic liver CT protocol at our institution includes precontrast, late arterial, portal venous, and delayed phases. After precontrast scanning, patients received an intravenous injection of 2.0 mL/kg of iodinated contrast medium, followed by a 20 mL saline bolus at a fixed injection duration of 30 s. Using the bolus-tracking method, late arterial phase images were acquired 18 s after the attenuation value reached 100 HU in the abdominal aorta. The portal venous and delayed phases began with delay times of 30 and 150 s after the late arterial and portal venous phases, respectively. The scanning parameters were as follows: 120 kV; 240 mAs; rotation time, 0.5 s; beam pitch, 2; and slice thickness, 3–5 mm.

### Image preprocessing

**Figure 5:**
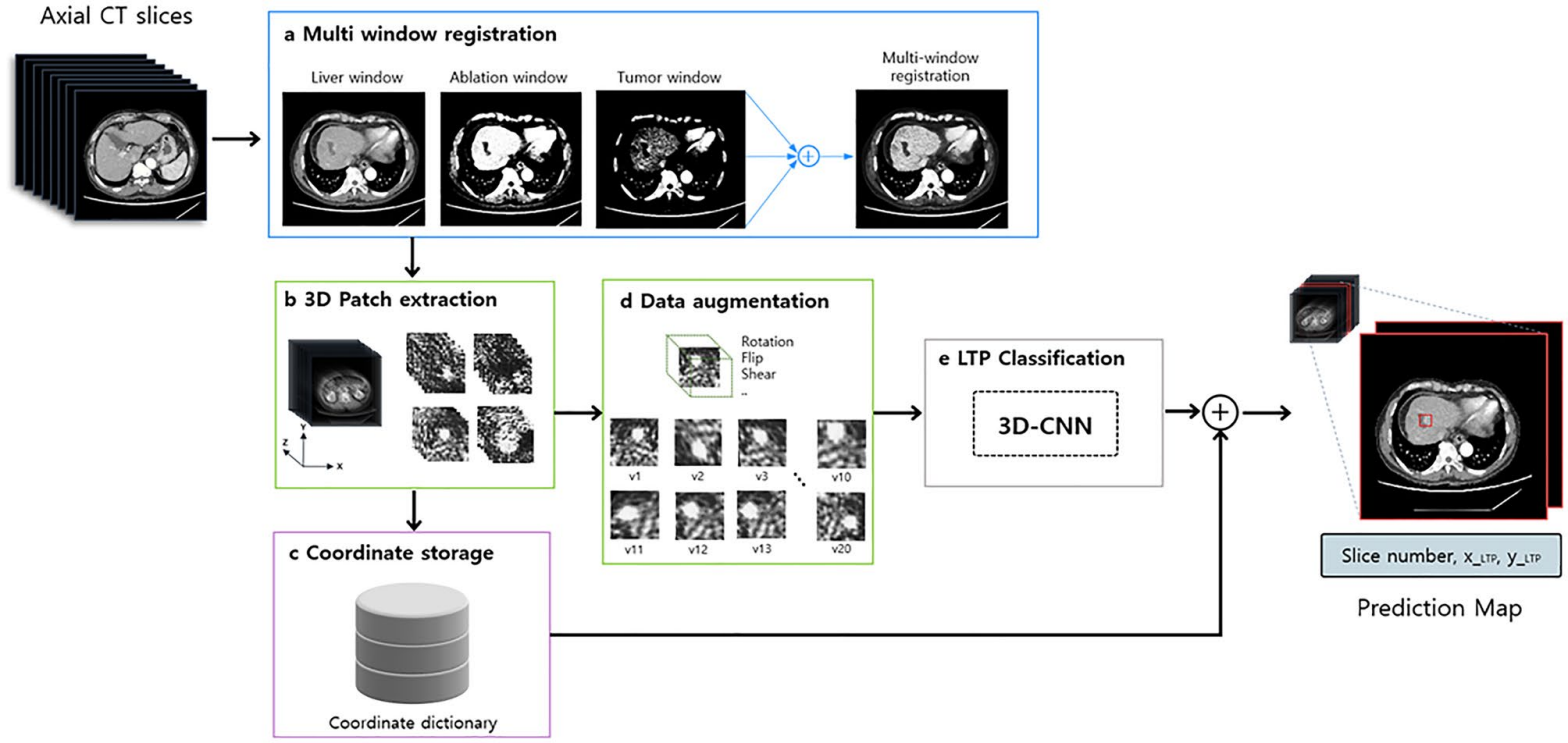
Schematic representation of image preprocessing/data augmentation steps and 3D-CNN model for LTP classification. The system includes a pipeline of the tumor region proposal technique.

We preprocessed DICOM files to extract 3D patches from the CT volume voxel to define candidates for LTP classification. Full-resolution CT images (512 × 512 pixels) with different predefined window levels and widths were spatially co-registered to obtain high LTP conspicuity images (Fig. 5). The conspicuity of LTP in liver RFA was maximized in three 8-bit grayscale images with different window settings and registration into the following RGB images: liver window (window level (WL) = 60, window width (WW) = 400) for the red channel, ablation window (WL = 80, WW = 60) for the green channel, and tumor window (WL = 120, WW = 40) for the blue channel. A multichannel registration was applied because the network learns to focus on the ablated zone, as ablated tissues have lower HU densities compared with the surrounding non-ablated liver parenchyma in CT images. Furthermore, the tumor window enhanced the appearance of LTP near the ablated region.

### CNN model and method

#### Patch extraction

The proposed CNN model utilizes 3D patches extracted from three-channel CT imaging to detect LTP. Three-dimensional volumetric voxels were converted to stacked 2D pixels (image set) to obtain the center coordinate of the LTP, p = (x,y,z), which were annotated by one radiologist (S Lim) in the right anterior superior space of the open-resource software package, 3D Slicer (Version 4.10.2, available: http://www.slicer.org).

For each positive candidate, a 2D axial view covering a neighborhood of 32 × 32 pixels containing the (x,y) coordinate in the z^th slice was obtained. A false positive reduction stage was constructed by stacking eight adjacent slices centered on z, resulting in a patch measuring 32 × 32 × 8 (Fig. 5). Multiple patches were resampled along the X and Y axes to contain p. Negative class candidates in the area outside a sphere of radius (r = 15) centered at the top were sampled with a 32 × 32 × 8 patch for patients with LTP after RFA. Patients who underwent RFA with no incidence of LTP were also considered to incorporate a wider spectrum of patients for LTP screening; for these cases, random 32 × 32 × 8 patches were sampled to be added to the negative candidate dataset. An example of the extracted patches is shown in Fig. 5.

#### CNN architecture

**Figure 6:**
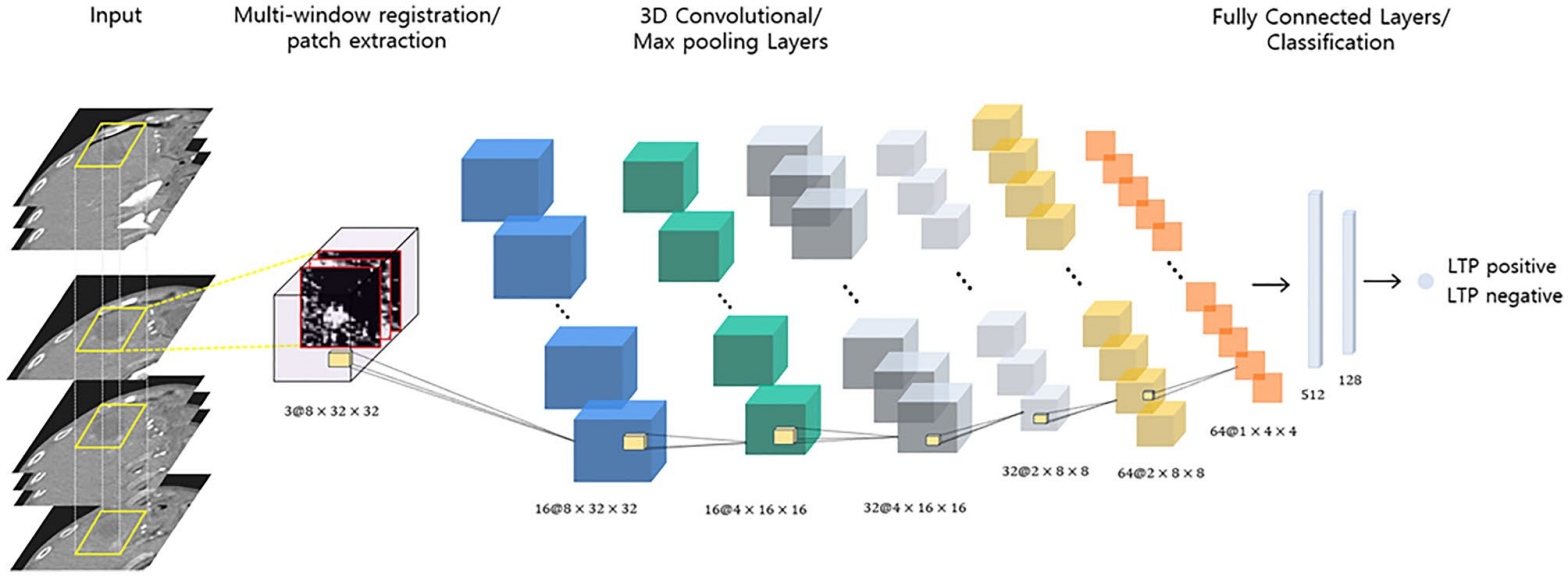
Illustration of the proposed 3D-CNN architecture. The network contains three convolutional layers, two max-pooling layers, and two fully connected layers. The number below each layer represents the feature map size after convolution/pooling.

The proposed CNN model inputs 3D patches of three-channel CT imaging for LTP detection. The proposed 3D CNN is illustrated in Fig. 6. The input of the network was a 32 × 32 × 8 patch. After the input layer, the next three layers were convolution layers followed by max-pooling layers (Fig. 6). For the convolutional layers, we applied a 3D kernel measuring 3 × 3 × 3 and a max pooling layer measuring 2 × 2 × 2. The first convolutional layer comprised 16 feature maps measuring 8 × 32 × 32; the second convolutional layer comprised 32 feature maps measuring 4 × 16 × 16, and the third convolutional layer comprised 64 feature maps measuring 2 × 8 × 8. Each convolutional layer produced multiple 3D outputs, and the max pooling layers reduced the size of the patches by half in all three axes. The last two consecutive fully connected layers contained 512 and 128 output units, followed by a final sigmoid layer. Rectified linear units were used in the convolutional and fully connected layers.

#### Tumor region proposal network

To identify the tumor-positive predicted region, we built a pipeline to automatically produce a bounding box localization of the pathological regions using a 3D-CNN trained for classification. Before predicting the test set, the (x,y,z)-center coordinates of the generated patches were stored in a dictionary during the image processing phase. In the prediction stage of the test set, image patches with classification probabilities above the optimal threshold value that were selected based on sensitivity and specificity were summarized. The corresponding (x,y,z) coordinates of the positively predicted image patches were retrieved from the coordinate dictionary, as shown in Fig. 5, where the corresponding rectangular regions of interest were drawn on the axial slice of the entire CT scan. Training took less than 10 min on two CPUs (Xeon E5-2630 2.2 GHz, Intel, Santa Clara, CA) with NVIDIA Quadro P5000 GPUs (NVIDIA Corp., Santa Clara, CA).

#### Experimental setup

As our method focuses on the detection of LTP from whole abdominal CT images automatically, large numbers of patches (as training samples) are required to facilitate model training. Positive class patches were extracted such that they contained the lesion of interest; in this study, 49 LTP lesions were observed in 34 patients. From the 49 LTP lesions, 36, 5, and 8 center coordinates composing the tumor regions for the training, validation, and test sets, respectively, which were annotated by experts, were randomly selected for positive patch extraction. For the negative class, the patches were extracted at the per-patient level considering two situations as follows: (1) Patches extracted from LTP positive patients that were sampled from normal regions outside of a sphere of radius (r = 15) at the LTP center point. A total of 34 cases were randomly divided into three sets: training (21 cases), validation (5 cases), and test (8 cases) for normal region extraction; (2) Normal regions from patients who underwent RFA but had no indication of LTP. A total of 40 cases were randomly divided into three sets: training (27 cases), validation (5 cases), and test (8 cases). Extracted patches that composed the test dataset were manually reviewed by a radiologist (S Lim) for the presence or absence of LTP and to ensure data validation quality.

As less than 2% of the voxels belonged to the LTP, whereas more than 98% of the voxels were non-LTP regions, the binary classifier must manage the high data imbalance problem, where randomly selected patches can easily cause the model to be overwhelmed by non-LTP features. Therefore, for each tumor, 20 patches containing the LTP center coordinates were sampled (patch extraction method) and random augmentations, including flipping, shift, sheer, zoom in/out, and rotation were applied on the axial plane. By augmenting the existing data samples, the generalizability of our network to the positive class can be increased. Furthermore, as the negative class comprised more diverse characteristics/regions (aorta, kidney, rib bone, etc.) compared with the positive patches, we extracted twice the number of patch samples (n = 40) for each patient case. The patient and data distributions are provided in Table 3.

**Table 3:** Patient and data distribution of 74 patients used for training and testing.

	Training	Validation	Test	Total
**LTP group (n = 34)**				
Tumor/LTP (positive case)				
No. of tumors	36	5	8	49
No. of patches	720	100	160	980
Non-Tumor/LTP (Negative class)				
No. of tumors	21	5	8	34
No. of patches	840	200	320	1360
**No LTP group (n = 40)**				
Non-Tumor/LTP (Negative class)				
No. of tumors	27	5	8	40
No. of patches	1080	200	320	1600

LTP, local tumor progression.

### Statistical analysis

Statistical analysis was performed using MedCalc software (MedCalc 19.2.1; MedCalc, Mariakerke, Belgium). A *P* value less than 0.05 was considered to indicate a statistically significant difference. Descriptive statistics were calculated for our population. The normality of the variables was analyzed using the Shapiro–Wilk test. Variables were compared, as appropriate, using Student’s t-test, χ^2^, or Fisher's exact test.

In our experiments, we measured the following performance metrics of the 3D-CNN prediction: accuracy, sensitivity (recall), specificity, positive predicted value (precision), average precision, and area under the receiver operating characteristic curve (AUC). The accuracy was determined for the 3D-CNN model with the optimal threshold that was selected based on the average sensitivity and specificity of the validation set. Furthermore, the receiver operating characteristic (ROC) curves and precision–recall curves were plotted. The precision–recall curve shows the relationship between the positive predictive value (PPV, precision) and sensitivity (recall), providing complementary information to the ROC curves, which are especially sensitive to an imbalanced dataset. All metrics were computed using the Scikit-learn machine-learning module (0.21.3) available in Python.
